# Space-time distribution of urinary incontinence outpatient production in men, Brazil, 2010-2019

**DOI:** 10.1590/S2237-96222022000200025

**Published:** 2022-09-16

**Authors:** Fernanda Vieira Nicolato, Alfredo Chaoubah, Maria do Socorro Lina van Keulen, Marcio Fernandes dos Reis, Graziela Liebel

**Affiliations:** 1 Universidade Federal de Juiz de Fora, Programa de Pós-Graduação em Saúde Coletiva, Juiz de Fora, MG, Brazil; 2 Universidade do Vale do Itajaí, Programa de Pós-Graduação em Saúde e Gestão do Trabalho, Itajaí, SC, Brazil

**Keywords:** Urinary Incontinence, Men’s Health, Time Series Studies, Geographic Mapping

## Abstract

**Objetive:**

To estimate the temporal trend and spatial distribution of urinary incontinence outpatient production in men in Brazil.

**Methods:**

This was an ecological time series study of Brazil and its regions, from 2010-2019, using data from the Brazilian National Health System Outpatient Information System. Prais-Winsten regression was used to analyze the temporal trend in Brazil as a whole and by region. The autoregressive integrated moving average model was used to forecast the trend until 2024.

**Results:**

In 2010, 3,457 outpatient appointments for urinary incontinence in men were registered, rising to 16,765 in 2019, revealing a rising temporal trend [annual percentage change = 50.37%; 95% confidence interval (95%IC) 37.54;63.62]; and a forecast of growth for the period 2020-2024 (final ARIMA model: 1, 1, 0). The spatial distribution of urinary incontinence rates varied between the country’s macro-regions.

**Conclusion:**

There was a rising temporal trend in urinary incontinence outpatient production in men in Brazil between 2010-2019 with growth forecast until 2024. The highest rates occurred in the Southeast region and the highest increase was found in the Southern region.

**Study contributions**

**Main results**

A rising temporal trend was found in urinary incontinence outpatient production among men in Brazil from 2010-2019, as well as a forecast of growth until 2024. The highest rates occurred in the country’s Southeast region, while the greatest increase occurred in the Southern region.

**Implications for services**

The increase in demand for urinary incontinence outpatient care shows the need for more public policies related to men’s health, especially for prevention and defining strategies for the treatment of this health condition.

**Perspectives**

Improving access to diagnosis and treatment of urinary incontinence in the Brazilian National Health System, in Brazil’s different regions, and development of further research in this area will enable quality care for incontinent men.

## Introduction

Urinary incontinence (UI), defined as the unintentional passing of urine, is a recurrent health problem that can affect individuals of any age, sex or socioeconomic status.^1^ ^,^ ^2^ However, there is great variation in UI prevalence, depending on event definitions, urinary complaints, age and sex.^3^

Although UI prevalence is about twice as high in women, men are also frequently affected by the disease, although their epidemiological UI profile has not been investigated to the same extent as that of women,^3^ so that the need exists to evaluate its prevalence in males due to its effects on the quality of life of affected people, such as social isolation, low self-esteem, depression and anxiety.^4^ ^-^ ^6^

Urine leakage is a common complaint in elderly people, with age being an important UI risk factor.^3^ It is estimated that there will be an increase in UI prevalence as the population becomes older,^7^ making it necessary to develop health interventions targeting this segment of the population.^7^ ^,^ ^8^

Apart from age, prostatectomy is another UI risk factor in males.^3^ ^,^ ^4^ ^,^ ^6^ Prostate cancer screening results in increased cancer case detection and consequently also results in an increase in males seeking health care for UI,^9^ hence it must be taken into consideration that UI will lead to greater demand for health service care in the future, requiring the planning of prevention actions, professional training and treatment for this specific condition being available.^10^

Our analysis of UI outpatient production in Brazil over ten consecutive years and forecasting future trends in the demand for outpatient procedures related to UI will be relevant for Brazilian National Health System (*Sistema Único de Saúde* - SUS) managers, to plan and implement actions to prevent and treat UI in men. This research aimed to estimate the temporal trend and spatial distribution of UI outpatient production in men in Brazil.

## Methods

We conducted an ecological time-series study of UI outpatient production in Brazil and the country’s five geographic regions - North, Northeast, South, Southeast and Midwest - covering the period from 2010 to 2019. Data were obtained from the Brazilian National Health System Outpatient Information System (SIA/SUS), available via the SUS Department of Information Technology (DATASUS) and retrieved between August and November 2021

We included the records of adult male individuals [aged 20 and over, available from the Brazilian Institute of Geography and Statistics (*Instituto Brasileiro de Geografia e Estatística* - IBGE)], receiving health care according to the Brazilian Clinical Protocol and Therapeutic Guidelines for Non-Neurogenic Urinary Incontinence^2^ and classified according to the International Statistical Classification of Diseases and Related Health Problems - 10^th^ Revision (ICD-10) - as per the following codes: R32 - Unspecified urinary incontinence; N39.3 - Stress incontinence; and N39.4 - Other specified urinary incontinence. The inclusion criteria were defined with the aim of providing the best reflection of the study’s target population.

The public health system data were retrieved and processed using TabWin version 4.1.5. They were later exported to Microsoft EXCEL® for tabulation and then exported to the Statistical Package for the Social Sciences (SPSS), version 21.0, in order to perform the statistical analysis.

Absolute frequency of UI outpatient production was stratified in age groups: 20-29; 30-39; 40-49; 50-59; 60-69; 70-79; 80 and over. We used the Kolmogorov-Smirnov test for normality, followed by the Mann-Whitney U test to check for differences between two of these age ranges - adult males (20-59 years old) and elderly males (60 years and above) - regarding outpatient production, taking significance less than 0.05 (p-value < 0.5); and the Kruskal-Wallis test, followed by Dunn’s post hoc test, with the aim of identifying significant (p-value < 0.5) differences in outpatient production between all the age groups. 

UI outpatient production rates were calculated based on population estimates for each of the country’s regions from 2010 to 2019, using the frequency of procedures per year as the numerator, and the estimated population of males over 20 years old as the denominator, multiplied by 100,000 inhabitants. The population information and intercensal estimates, resulting from the 2010 Demographic Census, were obtained from the IBGE website on October 29, 2021.

We used the methodology described by Antunes and Cardoso^11^ to analyze the temporal trend over the period 2010-2019; and we calculated annual percentage change (APC) and 95% confidence intervals (95%CI) to estimate the time series trend, based on the following formulae^11^

$$APC=\left[-1+10^{\beta 1}\right]*100\%$$

$$\mathrm{95}\%IC=\left[-1+10^{\beta 1mín}\right]*100\%;\;\left[-1+10^{\beta 1máx}\right]*100\%$$

where β1 is the slope (regression coefficient) of a linear regression model.

In the case of data representing a social phenomenon, generalized linear regression should be employed, whereby the Prais-Winsten method is the most widely used in this calculation.^11^

Prais-Winsten regression adjusts a model for dependent and independent variables in which error terms are autocorrelated. The most common process in which this happens is a first-order autocorrelation model, AR(1). Linear regression can be expressed as follows:

$$\gamma_{t}=\;\times_{t}\beta +\varepsilon_{t}$$

where the error terms satisfy:

$$\varepsilon_{t}={\rho \varepsilon }_{t-1}+\omega_{t}$$

The Prais-Winsten model is an estimator of generalized least-squares (GLS), and the method is derived from the AR(1) of the error term shown above. In order to check for serial autocorrelation in the data used, we performed the Durbin-Watson test, the null hypothesis of which is that serial autocorrelation is equal to 0.

We used the autoregressive integrated moving average model (ARIMA), based on the Box-Jenkins method,^12^ to forecast the temporal trend from 2020 to 2024. In the ARIMA model (p, d, q), "p" stands for the number of parameters in the autoregressive (AR) model, "d" stands for the degree of differentiation of the data series (I) for removing trend or seasonality in the data series, and "q" stands for the order of the moving average (MA).^12^ ^,^ ^13^ As such, the model developed to forecast the growth of UI outpatient production for men used p = 1, d = 1 and q = 0 as its parameters, according to the ARIMA model (1, 1, 0). 

We used the Akaike Information Criterion (AIC) to choose the most appropriate ARIMA model. AIC is a metric for measuring the quality of statistical models. The AIC-based model selection method considers the model with the lowest AIC to be the best model. We use the mean absolute error (MAE) to assess the final model fit. MAE measures the average distance between predicted values and observed values, and is the average of the prediction errors. The higher the MAE value, the worse the model is, because, on average, the predicted values are further away from the observed values.

The spatial distribution of the UI outpatient production rates was considered according to Brazil’s five geographic macro-regions. The maps were produced using the DATASUS system TabWin program. The years 2010, 2015 and 2019 were taken as the time frames. 

The study was based on secondary public domain data taken from the SIA/SUS system, available for access on the DATASUS website. It was therefore not necessary to submit the project to a Research Ethics Committee.

## Results

**Figure 1:**
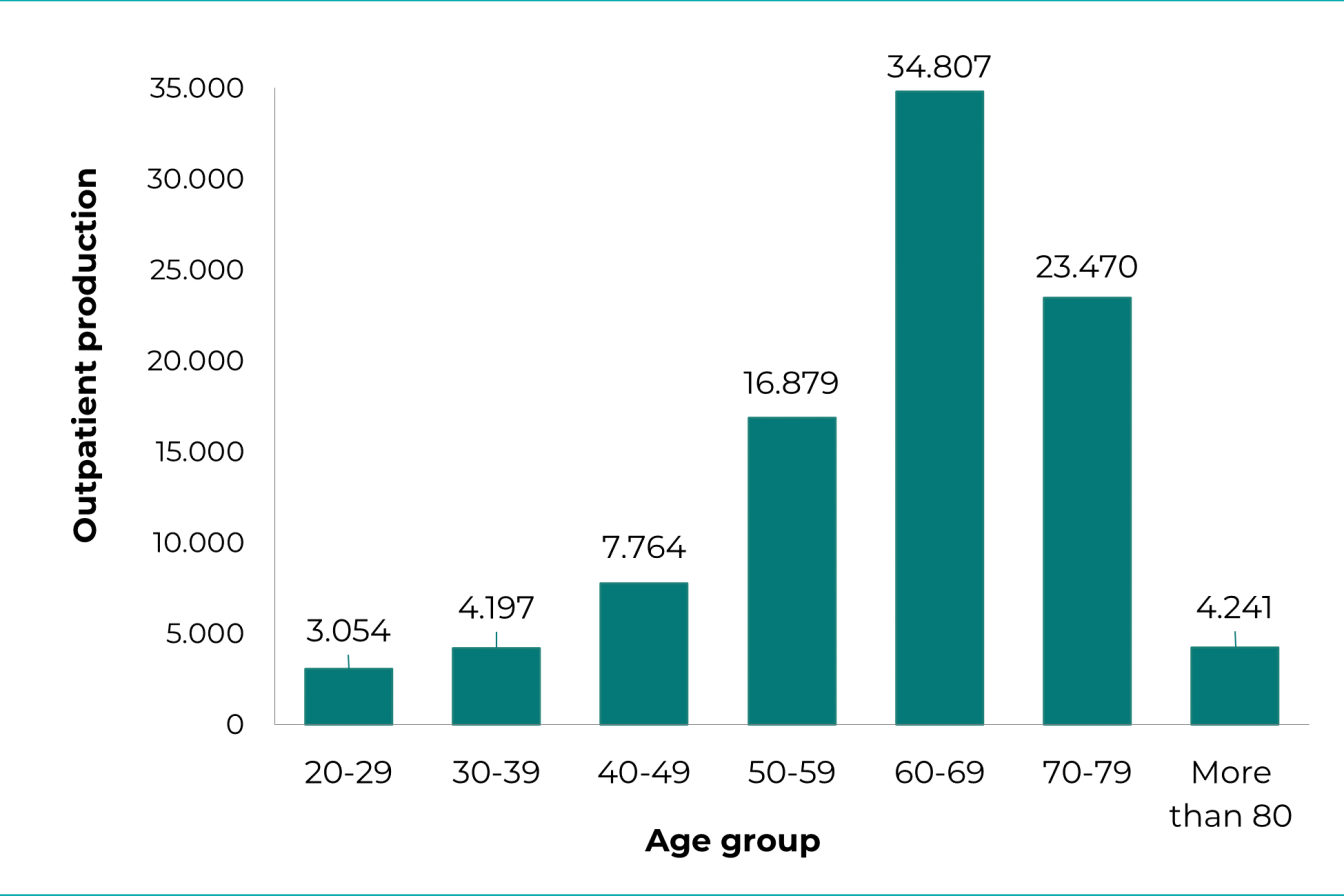
Urinary incontinence outpatient production in men by age group, Brazil, 2010-2019

Figure 1 shows the absolute frequency of UI outpatient production in men in Brazil, by age group, between the years 2010 and 2019. The highest frequency occurred in elderly men (60 years and over: 66% of outpatient procedures), when compared to absolute frequency in adult men aged 20 to 59 years (p-value = 0.000); and it was more expressive in the 60 to 79 age group, which accounted for 62% (p-value = 0.034).

In the study period, 2010 to 2019, the SUS recorded 94,418 UI outpatient procedures in men: in 2010, there were 3,457, rising to 16,765 in 2019. During the period between these dates, there was a 485% increase in UI outpatient production in Brazilian men, in addition to this time trend being shown to be rising and statistically significant, in Brazil as a whole (APC = 50.37%; 95%CI 37.54;63.62) and in each of its macro-regions: South (APC = 167.72%; 95%CI 122.37;221.46); North (APC = 100.64%; 95%CI 54.71;160.44); Midwest (APC = 46.35%; 95%CI 21.16;74.63); Southeast (APC = 45.00%; 95%CI 27.09;65.89); and Northeast (APC = 39.07%; 95%CI 2.53;69.71) (Table 1).

**Table 1:** Urinary incontinence outpatient production in men by macro-region, Brazil, 2010-2019

Region	2010	2011	2012	2013	2014	2015	2016	2017	2018	2019	2010-2019 (%)	APC^a^ (%)	95%CI^b^ min (%)	95%CI^b^ max (%)	Trend
**Outpatient production**															
North	50	83	37	48	167	225	164	332	342	810	1,620	101	55	160	↑
Northeast	1,017	235	969	745	938	922	1,085	1,275	1,741	1,475	145	39	3	70	↑
Southeast	2,109	4,099	4,831	3,989	4,720	9,439	8,947	8,584	9,404	11,070	525	45	27	66	↑
South	62	202	190	301	290	854	978	1,784	3,651	2,833	4,569	168	122	221	↑
Midwest	219	127	200	247	210	184	478	534	645	577	263	46	21	75	↑
**Brazil**	3,457	4,746	6,227	5,330	6,325	11,624	11,652	12,509	15,783	16,765	485	50	38	64	↑
**Growth rate**															
North	1.1	1.7	0.7	0.9	3.2	4.2	3.0	6.0	6.0	13.9	1,302	-	1.4	6.8	↑
Northeast	6.2	1.4	5.7	4.3	5.4	5.2	6.0	7.0	9.4	7.9	127	-	4.4	7.3	↑
Southeast	7.7	14.7	17.1	13.9	16.2	31.9	29.8	28.2	30.4	35.3	459	-	16.0	29.0	↑
South	0.7	2.1	2.0	3.0	2.9	8.4	9.5	17.0	34.3	26.3	3,986	-	2.7	18.5	↑
Midwest	4.8	2.7	4.1	5.0	4.1	3.5	9.0	9.9	11.7	10.2	215	-	4.3	8.7	↑
**Brazil**	5.5	7.5	9.6	8.1	9.4	17.1	16.9	17.8	22.2	23.2	419	-	9.4	18.1	↑

a) APC: *Annual percentage change*; b) 95%CI: 95% confidence interval; Rising trend (↑).

**Figure 2:**
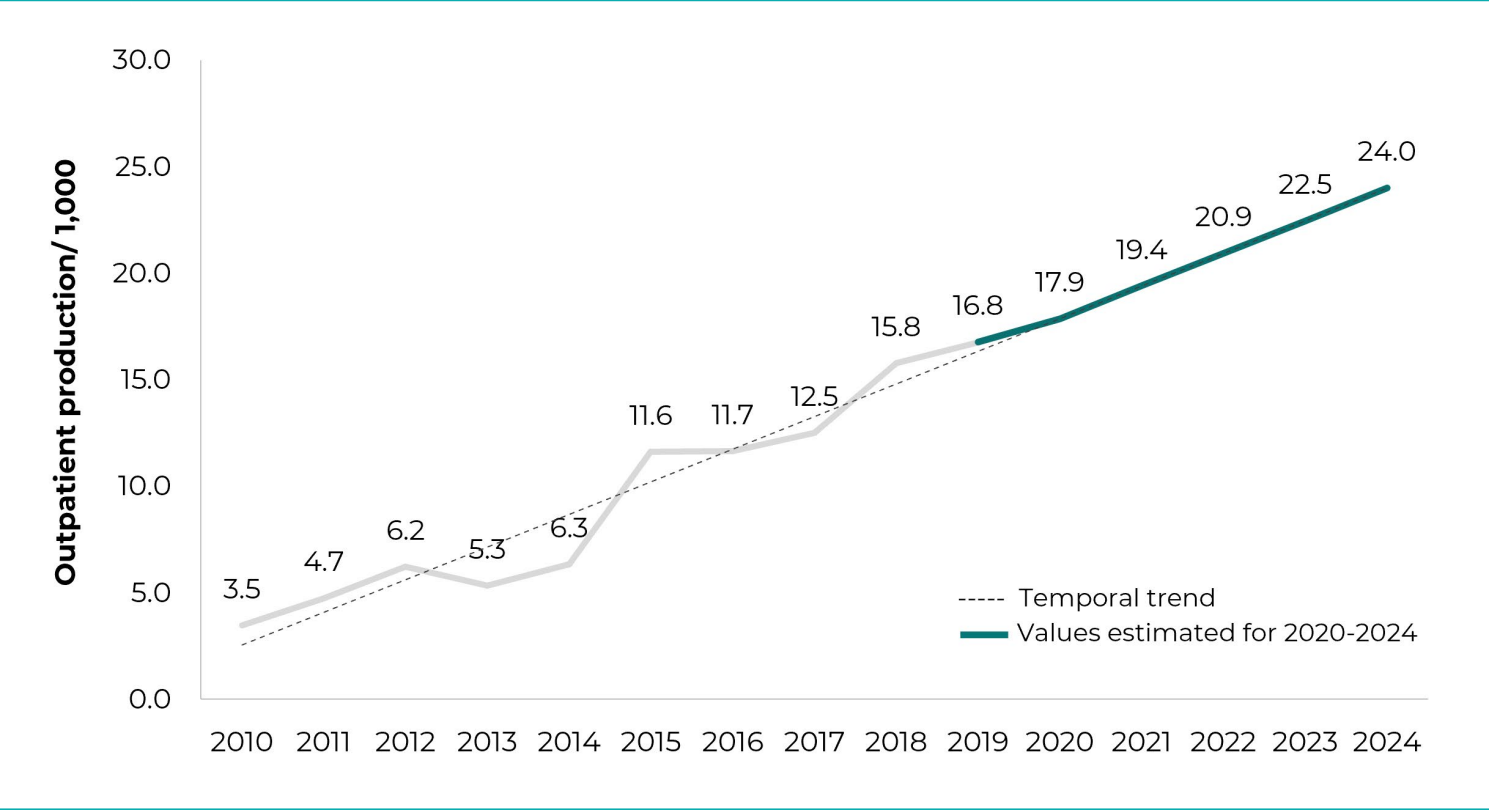
Time trend (2010-2019) and forecast (2020-2024) of urinary incontinence outpatient production in men, Brazil

Note: 2010-2019 - Brazilian National Health System outpatient production for urinary incontinence in men; 2020-2024 - period considered for forecasting outpatient production for urinary incontinence in men, using the ARIMA model (1, 1, 0).

Figure 2 presents the rising time trend line of UI outpatient production in men in Brazil between 2010 and 2019, as well as a forecast of growth in the following four years, 2020-2024, based on the final ARIMA model (1, 1, 0), with AIC = 168.08 and MAE = 1,529.825. 

**Figure 3:**
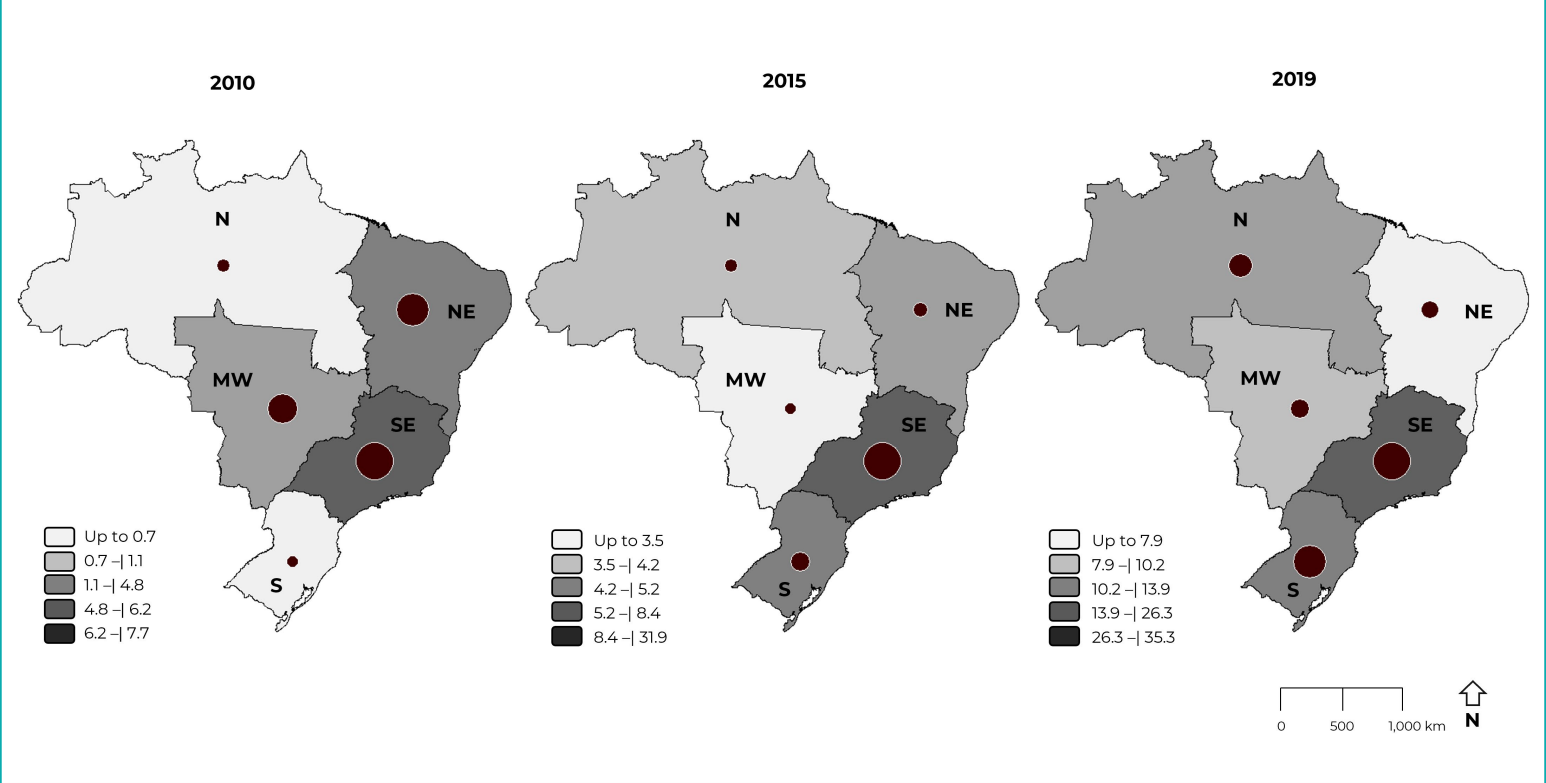
Spatial distribution of urinary incontinence outpatient production rates in men by macro-region, Brazil, 2010, 2015 and 2019

Legend: North (N); Northeast (NE); Southeast (SE); South (S); Midwest (MW).

The spatial distribution of the UI outpatient production rate in men (per 100,000 inhabitants) was analyzed for all regions (North, Northeast, South, Southeast and Midwest) of the country. In 2010, the regions with the highest rates were Southeast (7.7), Northeast (6.2), Midwest (4.8), North (1.1) and South (0.7); while for the year 2015, the highest rate corresponded to the Southeast region (31.9), followed by the South (8.4), Northeast (5.2), North (4.2) and Midwest (3.5); in 2019, the Southeast (35.3) and South (26.3) continued to present the highest rates, followed by the North (13.9), Midwest (10.2) and Northeast (7.9). Therefore, the spatial distribution of UI outpatient production in men in Brazil showed variation in the rates between the country’s geographic macro-regions: the highest rates corresponded to the Southeast, although the highest increase was found in the South (3.986%) (Figure 3).

## Discussion

The highest frequency of UI outpatient production in men in Brazil occurred among the elderly, especially those aged 60 to 79 years. UI outpatient production showed a rising temporal trend between 2010 and 2019, and this growth is expected to continue until 2024. The highest rates of UI outpatient production in men (per 100,000 inhabitants) corresponded to the Southeast region, although the Southern region showed the greatest upward trend over the period studied.

The results of our study confirm the findings of previous studies, which found higher UI prevalence in the elderly, pointing to age as a factor contributing to this increase.^3^ ^,^ ^7^ However, those studies found variable UI prevalence (%) in elderly men: 10.3; 11.8; 17.0; 30.5.^14^ ^-^ ^17^

According to the Portuguese Neurology and Urogynecology Association,^18^ it is estimated that about 35% of people over 60 years and 50% to 85% of elderly people living in institutions suffer from UI. It is therefore important to reflect on the high prevalence of UI in institutionalized elderly people.^19^ ^,^ ^20^

International studies show a level of UI prevalence that deserves attention, as well as a rising trend in rates.^10^ ^,^ ^21^ In a systematic review of global prevalence and economic burden of urgency UI, Milsom et al.^21^ estimated prevalence ranging from 1.8% to 30.5% in Europe, and from 1.7% to 36.4% in the United States, depending on age and sex.^21^ In 2008, approximately 348 million individuals worldwide experienced some degree of UI, with a projected 10.8% increase to 386 million in 2013 and a 21.6% increase to 423 million in 2018.^10^

Given these findings, it is expected that more men will require health care for UI and, consequently, that there will be an increase in demand for these health services. The results of this study indicate an increasing trend in UI outpatient production among men, in addition to predicting that it will continue to increase in forthcoming years. Moreover, this trend in outpatient production can be explained by the occurrence of post-prostatectomy UI, since increased UI is expected as more men undergo radical prostatectomy.^9^ ^,^ ^22^

We had difficulty in comparing the results of our analysis with data from the literature, given the scarcity of publications involving outpatient care for incontinent men. Notwithstanding, it is important to highlight that men require attention and care for UI,^4^ and, on a broader level, it is also important to discuss men’s health care in the context of the SUS. 

The National Policy on Comprehensive Men’s Health Care aims to improve their health conditions, seeking to reduce morbidity and mortality by rationally addressing risk factors and facilitating access to comprehensive care actions and services. As such, it is important to strengthen primary health care, including facilitating and ensuring access to and quality of men’s health care.^23^

The work of primary health care is essential for achieving better therapeutic and prognostic results in cases of incontinent people. At this level of care, it is important (i) to assess UI risk, (ii) to identify this health condition at its initial stage and (iii) to quickly and appropriately refer those diagnosed to specialized care.^2^ A study has shown that men negatively assess their first access to health services.^24^

However, our study relates to UI procedures for men performed at the specialized level of the SUS, given that this is outpatient care and not primary care, whereby it should be noted that referral is required in order to have access to this level of care, either from primary care or tertiary health care services. However, the availability of outpatient services reflects the regional disparities existing in Brazil, where barriers to access to primary health care can impact outpatient care and the loss of the ability of the SUS to provide this level of care.

Our study revealed that the highest rates of UI outpatient procedures in men occurred in the Southeast region, and that the greatest increase in their rates occurred in the Southern region. Both these regions have better infrastructure and greater access to health services. Therefore, the results of the study show how essential it is to analyze barriers to accessing public health services, in order to understand, in greater depth, the use of these services by the population and their limitations, in the light of different regional contexts.^25^

In general, there are fewer and less diverse barriers to health care services located in the South and Southeast regions. A survey on health service users’ beliefs as to their not having health problems highlighted reports of poor health service availability, as well as difficulties in accessing them, especially in the North, Northeast and Midwest regions of Brazil.^25^

Brazil is marked by profound regional inequalities, attributed to historical legacies. The territorial configuration of the SUS not only expresses but also reproduces these inequalities^26^ and, in order to address them, it is essential to develop public health policies that respect regional specificities and the health care needs of men.

The limitations of this study relate to the use of secondary data, which may represent only a portion of the outpatient production regarding incontinent men. Furthermore, these data are susceptible to errors. In addition, taking the country’s regions as the units of analysis may hide important inequalities within them, which could possibly come to light if large regions such as these were subdivided into smaller units. Given that this is an ecological study, another limitation could be the so-called "ecological fallacy", when inferences are made about individuals based on aggregate data for a group.

This study found a rising temporal trend in UI outpatient production in men in Brazil from 2010 to 2019, with a forecast of growth until 2024. The spatial distribution of these services showed variation between Brazil’s geographic regions, with the highest rates found in the Southeast region and the highest increase in the Southern region. The need therefore exists to implement actions to prevent and treat this health condition that meet the needs and specificities of the affected population. Although indirectly, the results of this study, as well as other contributions, point to the need for more public policies targeting men’s health, especially with regard to prevention and definition of strategies resulting in the treatment of UI
